# Characterization of advanced Parkinson’s disease in Germany: results of the non-interventional OBSERVE-PD study

**DOI:** 10.1186/s42466-022-00176-x

**Published:** 2022-03-15

**Authors:** David J. Pedrosa, Florin Gandor, Wolfgang H. Jost, Carolin Arlt, Koray Onuk, Lars Timmermann

**Affiliations:** 1 Klinik für Neurologie, Universitätsklinikum Gießen and Marburg, Marburg site, 35043 Marburg, Germany; 2 Neurologisches Fachkrankenhaus für Bewegungsstörungen/Parkinson, Kliniken Beelitz, 14547 Beelitz-Heilstätten, Germany; 3 Klinik für Neurologie, Otto-Von-Guericke-Universität Magdeburg, 39120 Magdeburg, Germany; 4 Parkinson-Klinik Ortenau, 77709 Wolfach, Germany; 5 AbbVie Deutschland GmbH & Co. KG, 65189 Wiesbaden, Germany; 6 AbbVie Inc., North Chicago, IL 60064 USA

**Keywords:** Advanced Parkinson’s disease, Treatment, Movement disorder centers, Germany

## Abstract

**Background:**

Parkinson’s disease (PD) is a progressive, neurodegenerative disorder. In the advanced stages it can result in severe disability despite optimal treatment. Data suggests heterogeneous classification of PD stages among physicians in different countries. The purpose of the OBSERVE-PD study was to evaluate the proportion of patients with advanced PD (APD) according to physicians’ judgments in an international cohort.

**Methods:**

A cross-sectional, observational study was conducted in 18 countries. Data were collected during a single patient visit. Demographic data, disease status, current medical treatment, and quality of life were evaluated for the German cohort and compared to the international cohort. Potential prognostic factors of physicians’ classification of APD in the German and international cohorts were identified using logistic regression.

**Results:**

In total, 177 German and 2438 international patients were enrolled. 68.9% of the German and 50.0% of the international patients were classified by physicians as APD. Despite similar demographics and comparable disease severity, motor fluctuations (odds ratio [OR], 49.7; 95% confidence interval [CI], 8.5–291.9) and current device-aided treatment (OR 8.7; CI 5.5–13.8) showed the strongest association to physicians’ classification of APD in the German and the international cohorts, respectively. The number of different oral anti-Parkinson-medications showed opposed associations with APD-classification between the international (OR 1.19; CI 1.03–1.37) and German (OR 0.46; CI 0.18–1.18) cohort. Although 58.2% of the German patients diagnosed with APD were considered eligible for device-aided treatment, only 40.8% actually received it.

**Conclusions:**

This study highlights the challenges in the recognition and the effective management of APD in Germany and emphasizes the necessity of complying with standard diagnostic criteria for identification of patients with APD. Therapeutic approaches differed internationally, with a tendency in Germany towards a more complex oral medication regimen for patients with APD. In view of similar quality of life and disease status in both cohorts, our findings may prompt further exploration of parameters for disease classifications, and consideration of optimal treatment strategies.

**Supplementary Information:**

The online version contains supplementary material available at 10.1186/s42466-022-00176-x.

## Background

Parkinson’s disease (PD) is the second most common neurodegenerative disorder [1]. With increasing disease duration, PD patients may develop severe disability despite patient-specific treatment [1, 2]. The progressing degeneration of dopaminergic neurons in the substantia nigra, the pathophysiological hallmark of this disease, requires dopamine substitution for symptom control. While dopaminergic medication usually provides good symptom control in the initial stages of disease, its effectiveness may deteriorate over time, including an increased sensitivity to subtle fluctuations in the drugs’ plasma levels, which ultimately narrows their therapeutic window. In consequence, hypo- and hyperkinetic motor fluctuations and non-motor fluctuations may emerge, and have been associated with the duration of levodopa treatment [3–5]. Furthermore, the development of symptoms irresponsive to conventional dopaminergic treatment add to the decrease in quality of life [6, 7].

There is no universally accepted consensus on how to define stages of PD considering motor and non-motor symptoms [8], although a patient’s disease stage may be a determining factor for optimal treatment. As the disease progresses, patients may experience an increased amplitude and frequency of fluctuations between periods of good and poor symptom control. Although device-aided treatments, such as deep brain stimulation, subcutaneous apomorphine, or intestinal levodopa infusion, are efficacious options in treating fluctuations, the decision on when to recommend and initiate device-aided therapy differs according to the physician’s staging of the disease.

The purpose of the OBSERVE-PD study was to evaluate the proportion of patients with advanced PD (APD) according to physician’s judgment and to compare demographic data, current medical treatment, disease status, and quality of life between patients classified as APD versus those classified as non-APD. The study furthermore assessed the treating physician’s judgement on the eligibility for device-aided therapies. Observe-PD was conducted in 18 countries, recruiting patients at movement disorder centers and clinics (MDCs). In this work, we extracted data of the German cohort comparing demographics, disease status, and quality-of-life scores of patients classified APD versus non-APD. Our objective was to identify factors supporting physicians’ decision for classifying PD as advanced, and comparing the findings from the German cohort with the international cohort.

## Methods

### Study population

OBSERVE-PD was an observational, cross-sectional, non-interventional, multi-center study with 2615 patients conducted in 18 countries across different geographic regions between February 2015 and January 2016. The study design has been reported previously [9]. In brief, adult patients diagnosed with PD according to the UK Parkinson’s Disease Society Brain Bank criteria [10] who attended a routine clinical visit or were admitted to an MDC were recruited. Data were collected as part of routine care during one single visit and consisted of demographics and disease history (disease duration, motor fluctuations, referral history and disease stage), including previous and current treatments (type and number of current treatments, treatment response, form of application and eligibility for device-aided treatment). Additionally, site and physician characteristics were collected.

The study was approved by local ethics committees in all participating countries and was conducted in accordance with the ethical principles of the Declaration of Helsinki. Before inclusion, all participants signed a patient authorization or informed consent for use and disclosure of their personal health information.

### Disease status and quality of life questionnaires

Patient quality of life and disease status were evaluated by physicians with the Unified Parkinson’s Diseases Rating Scale (UPDRS parts II-IV) [11], the modified Hoehn and Yahr stage [12], and the Non-Motor Symptoms Scale for Parkinson’s Disease (NMSS) questionnaire [13]. To evaluate quality of life, patients completed the 8-item Parkinson’s Disease Quality of Life questionnaire (PDQ-8) [14].

Along with their subjective assessment of the PD stage (advanced vs not advanced), physicians completed an APD questionnaire developed by an international panel of experts on movement disorders using the Delphi method [15–17]. This questionnaire comprises 11 questions for the assessment of APD, with patients classified as advanced when any criterion is fulfilled (cumulative classification).

### Statistical analysis

All enrolled patients fulfilling the selection criteria and with a physician’s diagnosis on PD stage were included. Descriptive statistics were conducted for quantitative and qualitative variables in the German and international cohort and separately for those with and without APD.

For the German subpopulation two-sample *t* tests were performed to assess potential differences in disease status and quality of life scores between these subgroups. Cohen’s kappa was calculated as a measure for the alignment of the physicians’ subjective assessments with the cumulative Delphi classification as well as with the single responses to each of the 11 questions of the Delphi questionnaire.

Multivariable logistic regression models were applied for both cohorts to investigate prognostic factors (patients demographics, PD history and treatment, dichotomized Delphi criteria, physician/institution characteristics) on physicians’ APD classification. A backward selection procedure was applied, including a fivefold cross-validation to determine the average predictive performance quantified by the Area Under the Receiver Operating Characteristic (AUROC). The set of variables with the maximal average AUROC estimates across all backward selection steps was chosen. Missing data were imputed by a regression-based single imputation method.

All statistical analyses were conducted with SAS® 9.4 (SAS Institute, Cary, NC, USA).

## Results

### German cohort: comparison of APD and non-APD patients

In total, 177 patients were enrolled in Germany for the OBSERVE-PD study. Half of the patients were recruited from MDCs in public hospitals (50.0%), 17.1% from MDCs in university hospitals and 32.9% from MDCs in other institutions, by either general neurologists (23.2%), movement disorder specialists (33.9%) or physicians with multiple specialties (42.9%). Physicians classified more than two-thirds of the patients in an advanced stage of PD (n = 122 [68.9%]). Table 1 provides an overview of demographic data and disease characteristics of patients classified as APD and non-APD. There was a higher percentage of male patients in the non-APD versus APD group (67.3% vs 57.4%). Patients in the APD group had a longer disease duration (10.2 vs 3.1 years). The percentage of patients requiring caregiver support and experiencing motor fluctuations was higher in the APD cohort.

**Table 1:** Patient characteristics and PD related variables in the German cohort

	Determination according to physician's judgment						Total		
	APD			Non-APD					
	n	n_miss_	Mean ± SD/n (%)	n	n_miss_	Mean ± SD/n (%)	N	n_miss_	Mean ± SD/n (%)
Sex, male	122	0	70 (57.4)	55	0	37 (67.3)	177	0	107 (60.5)
Living at home, yes	122	0	120 (98.4)	55	0	54 (98.2)	177	0	174 (98.3)
Required caregiver support, yes	121	1	80 (66.1)	55	0	19 (34.5)	176	1	99 (56.3)
Age at patient visit, years	122	0	67.9 ± 9.1	55	0	70.4 ± 9.0	177	0	68.7 ± 9.1
Time since diagnosis of PD, years	119	3	10.2 ± 6.0	53	2	3.1 ± 2.8	172	5	8.0 ± 6.2
Motor fluctuations, yes	122	0	104 (85.2)	55	0	6 (10.9)	177	0	110 (62.1)
Duration of motor fluctuations^a^, years	100	4	3.5 ± 3.0	6	0	2.1 ± 1.7	106	4	3.4 ± 2.9
Referral to MDC, yes	122	0	112 (91.8)	55	0	46 (83.6)	177	0	158 (89.3)
Time since referral to MDC ^b^, years	109	3	3.6 ± 2.9	46	0	1.4 ± 2.0	155	3	2.0 ± 3.2

APD, advanced Parkinson’s disease; MDC, movement disorder center; n_miss_: number of missing values; PD, Parkinson’s disease

^a^Restricted to patients with motor fluctuations, ^b^Restricted to patients referred to MDC

In the German cohort, the APD classification relying on physicians’ judgement and the APD classification based on the Delphi method showed a fair consensus (Cohen’s kappa: 0.243, Additional file 1: Table S2). The vast majority of patients reported at least one comorbidity (APD, 95.1% vs non-APD, 85.5%; Additional file 1: Table S1) with subjective cognitive dysfunction (59.8% vs 50.9%), hypertension (36.9% vs 49.1%), and depressive symptoms (23.8% vs 16.4%) being the most prevalent.

Nearly all patients received dopaminergic treatment (APD, 99.2% vs non-APD, 96.4%). In comparison to non-APD, patients classified as APD were more frequently treated with oral levodopa (93.4% vs 76.4%), oral dopamine agonists (62.3% vs 34.5%) and catechol-o-methyltransferase inhibitors (27.0% vs 3.6%).

**Fig. 1:**
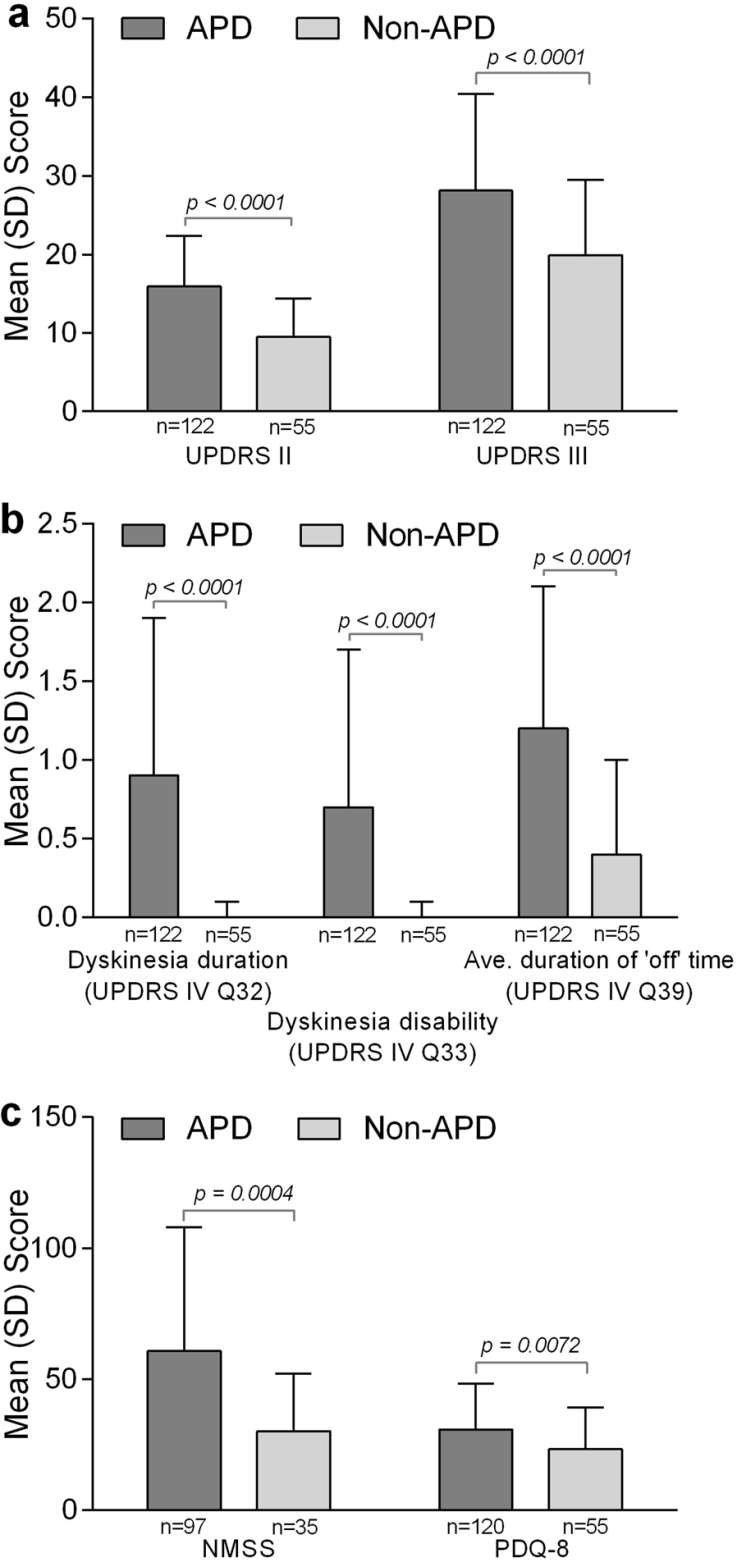
Disease status and quality of life scores for patients in the APD and non-APD subgroups. Disease status and quality of life scores for patients in the APD and non-APD subgroups as per **a** UPDRS parts II and III; **b** UPDRS part IV (dyskinesia duration, dyskinesia disability, and average duration of “off” time); and **c** the NMSS and PDQ-8. APD, advanced Parkinson’s disease; NMSS, Non-Motor Symptoms Scale for Parkinson’s Disease; PDQ-8, Parkinson’s Disease Questionnaire; Q, question; UPDRS, Unified Parkinson’s Disease Rating Scale

On average, UPDRS II, UPDRS III, UPDRS IV Question 32 (dyskinesia duration), and UPDRS IV Question 39 (average duration of “off” time) were significantly higher in patients with versus without APD (UPDRS II: mean: 15.9 ± SD: 6.5 vs 9.5 ± 4.9, *p* < 0.0001; UPDRS III: 28.1 ± 12.3 vs 19.9 ± 9.6, *p* < 0.0001, UPDRS IV Q32: 0.9 ± 1.0 vs 0.0 ± 0.1, *p* < 0.0001, UPDRS IV Q39: 0.7 ± 1.0 vs 0.0 ± 0.1, *p* < 0.0001). The mean total NMSS score was twice as high for patients in the APD subgroup versus the non-APD subgroup (60.7 ± 47.4 vs 30.1 ± 22.1, *p* = 0.0004). Significantly higher PDQ-8 scores were observed in the APD versus non-APD group (30.7 ± 17.5 vs 23.2 ± 16.0, *p* = 0.0072) (Fig. 1).

More than half of the APD group (58.2%) and a quarter of the non-APD group (25.5%) were deemed eligible for device-aided treatment, according to the judgment of the treating physicians (Table 2). Of these, 40.8% of the APD patients and 15.4% of the non-APD patients actually received device-aided treatment or treatment initiation was planned. The main reasons for patients not receiving or initiating a device-aided treatment were either indecisiveness or refusal of device-aided treatments altogether (Table 2).

**Table 2:** Device-aided treatment in the German cohort

Device-aided treatment	APD according to physician's judgment, n (%)		Total, N (%)
	APD	Non-APD	
Eligibility			
Yes	71 (58.2)	14 (25.5)	85 (48.0)
No	51 (41.8)	41 (74.5)	92 (52.0)
Status^a^			
Ongoing	23 (32.4)	2 (15.4)	25 (29.8)
Will begin device-aided treatment	6 (8.5)	0 (0)	6 (7.1)
No	42 (59.2)	11 (84.6)	53 (63.1)
Reason for no device-aided treatment^b^			
Age	2 (4.8)	0 (0)	2 (3.8)
Patient refusal	11 (26.2)	1 (9.1)	12 (22.6)
Patient needs more time to decide	25 (59.5)	7 (63.6)	32 (60.4)
Cognitive related issues	1 (2.4)	0 (0)	1 (1.9)
Psychiatric related issues	1 (2.4)	0 (0)	1 (1.9)
Comorbidities	1 (2.4)	0 (0)	1 (1.9)
Lack of caregiver/family support	1 (2.4)	0 (0)	1 (1.9)
Other	5 (11.9)	3 (27.3)	8 (15.1)

APD, advanced Parkinson’s disease

^a^Restricted to patients eligible for device-aided treatment, status missing for one non-APD patient

^b^Restricted to patients with no device-aided treatment although eligible, multiple entries possible

### Comparison of physicians’ APD classification in the German and international cohort

In contrast to the German cohort, most patients in the international cohort (n = 2438) were treated at university hospitals (63.7%) by movement disorder specialists (67.5%). Neither the demographics nor the disease status differed significantly between cohorts (Table 3). Furthermore, the number of patients experiencing “off” symptoms for more than 25% of the day were comparable (German cohort 78.0% vs international cohort 78.7%, *p* = 0.8076). Although more German patients were classified as APD than the international cohort (68.9% vs 50.0%, *p* < 0.0001), German patients received device-aided treatment less frequently (17.6% vs 22.6%, *p* = 0.1265). However, the latter difference did not reach statistical significance. Furthermore, the former were treated more frequently with ≥ 5 daily oral levodopa doses (42.6% vs 30.5%, *p* = 0.0008). Motor fluctuations occurred more frequently in the German cohort, although not significantly (62.1% vs 55.5%, *p* = 0.0834).

**Table 3:** Descriptive statistics and results of the regression models

Variable	International model		German model	
	Description	OR (95% CI)	Description	OR (95% CI)
Patient characteristic				
Age, mean ± SD, years	66.9 ± 9.9		68.7 ± 9.1	0.86 (0.78–0.96)
Sex, n (%)				
Male	1444 (59.2)	1.36 (1.03–1.8)	107 (60.5)	
Female	994 (40.8)	Reference	70 (39.5)	
Time since PD diagnosis, mean ± SD, years	7.7 ± 5.9	1.12 (1.08–1.16)	8.0 ± 6.2	1.34 (1.11–1.63)
UPDRS V: Modified Hoehn & Yahr Staging, mean ± SD	2.5 ± 0.8	2.04 (1.57–2.65)	2.7 ± 0.9	5.74 (1.34–24.58)
Treatment				
Number of current oral treatments, mean ± SD	2.0 ± 1.1	1.19 (1.03–1.37)	2.1 ± 1.0	0.46 (0.18–1.18)
Delphi 7. ≥ 5 times daily oral levodopa dosing				
Yes	740 (30.5)	2.02 (1.47–2.79)	75 (42.6)	
No	1687 (69.5)	Reference	101 (57.4)	
Current device-aided treatment (ongoing/about to start), n (%)				
Yes	549 (22.6)	8.68 (5.45–13.82)	31 (17.6)	
No	1883 (77.4)	Reference	145 (82.4)	
Dyskinesia				
UPDRS IV Q32: Dyskinesia (duration), n (%)				
0–25% of day	2074 (85.4)	Reference	152 (85.9)	
26–100% of day	355 (14.6)	1.44 (0.95–2.19)	25 (14.1)	
Delphi 4. ≥ 2 h of the day with troublesome dyskinesia				
Yes	406 (17)	1.60 (1.02–2.49)	26 (15.4)	
No	1985 (83)	Reference	143 (84.6)	
Motor symptom				
Motor fluctuations, n (%)				
Yes	1352 (55.5)	3.79 (2.78–5.17)	110 (62.1)	49.72 (8.47–291.91)
No	1086 (44.5)	Reference	67 (37.9)	Reference
UPDRS III: Motor examination, mean ± SD	25.7 ± 13.9		25.6 ± 12.1	1.09 (0.99–1.20)
Delphi 6. “off” time ≥ every 3 h				
Yes	546 (22.4)	1.56 (1.05–2.33)	40 (22.6)	
No	1888 (77.6)	Reference	137 (77.4)	
Daily living				
Patient requires help at home with daily activities				
Yes	1146 (47.3)	1.78 (1.30–2.43)	99 (56.3)	
No/not applicable	1275 (52.7)	Reference	77 (43.8)	
Patient lives at a nursing home/other				
Yes	56 (2.3)	4.64 (1.70–12.69)	5 (2.8)	
No	2379 (97.7)	Reference	172 (97.2)	
UPDRS II: Activities of daily living, mean ± SD	12.5 ± 8.2	1.04 (1.00–1.07)	13.9 ± 6.7	
Delphi 8. Moderate or severe limitation of activities of daily living				
Yes	854 (35.5)	1.38 (0.97–1.96)	70 (39.8)	
No	1550 (64.5)	Reference	106 (60.2)	
Health-related quality of life				
PDQ-8 score, mean ± SD	28.9 ± 19.8	1.01 (1.00–1.02)	28.4 ± 17.4	
Non-motor symptom				
NMSS score, mean ± SD	46.4 ± 39.1	1.00 (0.99–1.00)	52.6 ± 44.2	
Delphi 11. Moderate or severe psychosis				
Yes	100 (4.2)	2.61 (1.16–5.88)	7 (4.3)	
No	2290 (95.8)	Reference	154 (95.7)	
Delphi 5. Non-motor symptoms fluctuations				
Yes	938 (38.6)		80 (45.2)	4.31 (1.06–17.54)
No	1492 (61.4)		97 (54.8)	Reference
Physician characteristic				
Unit part of a dedicated referral network for PD, n (%)				
Yes	1714 (70.3)	1.40 (1.04–1.89)	127 (71.8)	
No	724 (29.7)	Reference	50 (28.2)	
Field of specialty, n (%)				
Neurologist (general)	448 (18.5)	Reference	41 (23.2)	Reference
Neurologist (movement disorder specialist	1636 (67.5)	0.43 (0.29–0.61)	60 (33.9)	0.19 (0.03–1.16)
Geriatrician	20 (0.8)	0.20 (0.02–2.78)	0 (0.0)	
Other	22 (0.9)	0.45 (0.09–2.32)	0 (0.0)	
Multiple	299 (12.3)	0.36 (0.21–0.61)	76 (42.9)	2.62 (0.44–15.45)
Site characteristic				
Site treatment algorithm, n (%)				
Yes	405 (16.8)	2.83 (1.94–4.12)	8 (4.5)	
No	2007 (83.2)	Reference	169 (95.5)	

CI, confidence interval; NMSS, Non-Motor Symptoms Scale for Parkinson’s Disease; OR, odds ratio; PD, Parkinson’s disease; PDQ-8, 8-item Parkinson’s Disease Questionnaire; SD, standard deviation; UPDRS, Unified Parkinson’s Disease Rating Scale

Only variables included in the international or German regression models are shown. Cells highlighted in gray refer to variables, which were not included in the regression model

When applying the suggested diagnostic criteria [15], the percentage of patients with APD was higher in Germany (84.8%) than in the international cohort (69.3%) (Table 4). 69.0% of German patients with PD deemed as non-advanced according to physician judgment would have fulfilled the Delphi criteria for APD, which was higher than in the international cohort (46.8%).

**Table 4:** Patients with APD classification

APD classification according to physician's judgment		Patients with APD classification by Delphi method				Total, n (%)
		Non-APD, n (%)	APD, n (%)	Not classified,^a^ n	Total classified, n	
International (without Germany)	APD	110 (9.0)	1109 (91.0)	1	1219	1220 (50.0)
	Non-APD	624 (53.2)	550 (46.8)	44	1174	1218 (50.0)
	Total	734 (30.7)	1659 (69.3)	45	2393	2438 (100.0)
Germany	APD	12 (9.8)	110 (90.2)	0	122	122 (68.9)
	Non-APD	13 (31.0)	29 (69.0)	13	42	55 (31.1)
	Total	25 (15.2)	139 (84.8)	13	164	177 (100.0)

*APD* advanced Parkinson’s disease

^a^Delphi method was not applied

Regression analyses indicated stronger effects in the occurrence of motor fluctuations on the physicians’ attribution of APD in the German (odds ratio [OR] 49.72; 95% confidence interval [CI] 8.47–291.91) than in the international cohort (OR 3.79; CI 2.78–5.17). Ongoing device-aided therapy was associated with physicians’ APD classification only in the international cohort (OR 8.68; CI 5.45–13.82) but not in the German cohort. The number of concurrently prescribed oral anti-PD medication was positively associated with assigning APD in the international cohort (OR 1.19; CI 1.03–1.37), while no such association was observed in the German cohort (OR 0.46; CI 0.18–1.18). In addition, Hoehn and Yahr stages and APD classification had a stronger association in the German (OR 5.74; CI 1.34–24.58) than in the international cohort (OR 2.04; CI 1.57–2.65).

The occurrence of non-motor symptoms showed no association with the physicians’ APD classification, neither in the international nor the German cohort. However, utilizing the Delphi criteria, criterion number 5 (non-motor symptom fluctuations) showed a significant association in the German cohort (OR 4.31; CI 1.06–17.54), and criterion number 11 (moderate or severe psychosis) in the international cohort (OR 2.61; CI 1.16–5.88).

## Discussion

This study presents the German data of the observational, multi-country, cross-sectional OBSERVE-PD study [9]. We identified differences between the German physicians’ judgements and the suggested criteria for the diagnosis of APD [15]. Furthermore, our results indicate that despite the more frequent assignment of APD in Germany, advanced treatment options such as device-aided therapies were initiated less commonly. In addition, the fact that 25.5% of non-APD patients were deemed eligible for device-aided treatment, of whom two patients eventually received a therapy escalation is an intriguing observation warranting further investigation. As pointed out by Fasano et al., enhanced patient-physician guidance may facilitate transition to initiation of device-aided treatment, a factor which may also play a key role in the patient cohort in Germany [9].

APD was generally well recognized in Germany. Consensus of physicians’ judgement and APD classification based on the Delphi method was fair in the German but moderate in the international cohort (German cohort: 0.243 vs. international cohort: 0.441). In general, Cohen’s kappa coefficients of all Delphi criteria items were lower in the German than in the international cohort. However, the highest agreement with physicians’ APD classification was found in both cohorts for the same items: “moderate/severe troublesome motor fluctuations” (0.315 vs. 0.425), “at least 5 times daily oral levodopa dosing” (0.344 vs 0.410), or “moderate/severe of limitation of ADL capacity” (0242 vs. 0.440). One may speculate the later release of the Delphi study [15] resulted in a potential knowledge gap of physicians regarding current diagnostic criteria. This could have had different implications according to medical system peculiarities. The German healthcare system profits from a higher number of specialized physicians who focus on the treatment of only certain disease groups, i.e. movement disorders in contrast to the more university-focused treatment of patients with PD in the international cohort. A somehow related yet different perspective of the apparent “anosognosia” for APD in subjects deemed as non-APD may turn towards different approaches, especially in view of the German medical infrastructure allowing more frequent patient assessments. Interestingly, the high hospitalization rates and oversupply with medical products are considered traditional flaws of the German healthcare system [18]. One may thus have anticipated a larger number of patients on device-aided treatments. Contrarily, the percentage of German PD-patients on or scheduled for device-aided treatment was lower than in the international cohort. Analyses in the Swiss cohort of the OBSERVED-PD study showed similar patient characteristics and APD frequency (69.4%) of the Swiss patients in comparison to the German patients [19]. However, despite these similarities and comparable healthcare systems in both countries, consensus of physicians’ judgement and APD classification based on the Delphi method (kappa: 0.480) as well as the percentage of patients with device-aided treatment was much higher (61.3%) in the Swiss cohort.

So why do German neurologists seem to be so cautious about diagnosing APD in general and specifically about recommending device-aided treatment options even in PD-patients who are deemed advanced? A comparison of disease severity revealed equal symptom burden in both cohorts when comparing overall clinical state including motor and non-motor features, ruling out disease-specific differences between cohorts. In contrast, patients’ indecisiveness and/or refusal were identified as possible causes for not administering device-aided treatments. Moreover, the aforementioned high density of qualified physicians in Germany [18] may allow for more frequent patient visits and for tailoring a more sophisticated oral therapy regime. Simply speaking, more fine-tuning of oral medications may have delayed the consideration of device-aided treatments by physicians and patients alike. However, in addition to the characteristic differences between the healthcare systems, there seemed to be also some notable differences in the parameters that were considered relevant for diagnosis of APD. As such, in Germany, data seem to suggest that physicians put a significantly higher emphasis on non-motor and motor symptoms, as well as the symptoms according to the Hoehn & Yahr scale, than in the international cohort. Importantly, results from both the German and the international cohorts show similar levels in quality of life despite the different treatment strategies, which indicates that German PD-patients are treated equally efficacious while agreeing to a more sophisticated regimen of oral medication. These findings might highlight the need for a thorough evaluation of patients’ individual treatment expectations to facilitate individual treatment recommendations [20, 21]. At this point, we advocate for a more detailed insight for distinct therapeutic options through future studies.

The generalizability of our results is subject to certain limitations. First, the sample size of the German study population is relatively small. Secondly, the suggested criteria for the diagnosis of APD [15] were published after data collection in the OBSERVE-PD study. Hence, and as already stated, awareness of this topic might have increased in the meantime, warranting further studies. Finally, our results might not be that easily translatable to the general PD population, since German patients were recruited from MDCs, which, owing to their treatment expertise, treat a higher proportion of patients with PD in the later stages of the disease.

## Conclusions

In summary, this study highlights the challenges in the recognition and the effective management of APD in Germany and emphasizes the necessity of complying with standard diagnostic criteria for identification of patients with APD. Therapeutic approaches differed internationally, with a tendency in Germany towards a more complex oral medication regimen for patients with APD. In view of similar quality of life and disease status in both cohorts, our findings may prompt further exploration of parameters for disease classifications, and consideration of optimal treatment strategies.

## Supplementary Information

**Additional file 1.** Supplementary Tables 1 and 2 with additional clinical information of German patients included in the observe-PD study. The two tables display the distribution of comorbidities and how many patients fullfilled the different criteria put forward at the Delphi study by Antonini and colleagues stratified for APD and non-APD subjects, respectively.

## Data Availability

AbbVie is committed to responsible data sharing regarding the clinical trials we sponsor. This includes access to anonymized, individual and trial-level data (analysis data sets), as well as other information (e.g., protocols and Clinical Study Reports), as long as the trials are not part of an ongoing or planned regulatory submission. This includes requests for clinical trial data for unlicensed products and indications. This clinical trial data can be requested by any qualified researchers who engage in rigorous, independent scientific research, and will be provided following review and approval of a research proposal and Statistical Analysis Plan (SAP) and execution of a Data Sharing Agreement (DSA). Data requests can be submitted at any time and the data will be accessible for 12 months, with possible extensions considered. For more information on the process, or to submit a request, visit the following link: https://www.abbvie.com/our-science/clinical-trials/clinical-trials-data-and-information-sharing/data-and-information-sharing-with-qualified-researchers.html.

## Abbreviations

APD: Advanced Parkinson’s disease
AUROC: Area under the receiver operating characteristic
CI: Confidence interval
MDC: Movement disorder center
NMSS: Non-Motor Symptoms Scale for Parkinson’s Disease
OR: Odds ratio
PD: Parkinson’s disease
PDQ-8: 8-Item Parkinson’s Disease Questionnaire
SD: Standard deviation
UPDRS: Unified Parkinson’s Disease Rating Scale
