# Supplementary material for: Characterization of advanced Parkinson’s disease in Germany: results of the non-interventional OBSERVE-PD study
Source: Neurol Res Pract. 2022 Mar 15;4:9. doi: 10.1186/s42466-022-00176-x (PMC8922845; doi:10.1186/s42466-022-00176-x)
Supplement: Supplementary file 1 — Additional file 1. Supplementary Tables 1 and 2 with additional clinical information of German patients included in the observe-PD study. The two tables display the distribution of comorbidities and how many patients fullfilled the different criteria put forward at the Delphi study by Antonini and colleagues stratified for APD and non-APD subjects, respectively. [file 42466_2022_176_MOESM1_ESM.docx]

**Characterization of Advanced Parkinson’s Disease in Germany: Results of the Non-interventional OBSERVE-PD Study**

David Pedrosa*, Florin Gandor, Wolfgang H. Jost, Carolin Arlt, Koray Onuk, Lars Timmermann

PD Dr David Pedrosa, Universitätsklinikum Marburg, Klinik für Neurologie, 35043 Marburg, Germany; david.pedrosa@staff.uni-marburg.de

Dr Florin Gandor, Kliniken Beelitz, Neurologisches Fachkrankenhaus für Bewegungsstörungen/ Parkinson, 14547 Beelitz-Heilstätten, Germany; Gandor@kliniken-beelitz.de

Prof Dr Wolfgang Jost, Parkinson-Klinik Ortenau, 77709 Wolfach, Germany; [w.jost@parkinson-klinik.de](mailto:w.jost@parkinson-klinik.de)

Carolin Arlt, AbbVie Deutschland GmbH & Co. KG, 65189 Wiesbaden, Germany; [carolin.arlt@abbvie.com](mailto:carolin.arlt@abbvie.com)

Koray Onuk, AbbVie Inc., North Chicago, IL 60064, USA; koray.onuk@abbvie.com

Prof Dr Lars Timmermann, Universitätsklinikum Marburg, Klinik für Neurologie, 35043 Marburg, Germany; Lars.Timmermann@uk-gm.de

***Corresponding author**

PD Dr David Pedrosa, Universitätsklinikum Gießen und Marburg, Standort Marburg, Klinik für Neurologie, 35043 Marburg, Germany

**Supplemental Table 1.** Comorbidities

|  | **APD according to physician's judgment** | |  |
| --- | --- | --- | --- |
|  | **APD** | **Non-APD** | **Total** |
|  | N (%) | N (%) | N (%) |
| Any comorbidity | 116 (95.1) | 47 (85.5) | 163 (92.1) |
| Cognitive dysfunction | 73 (59.8) | 28 (50.9) | 101 (57.1) |
| Hypertension | 45 (36.9) | 27 (49.1) | 72 (40.7) |
| Depression | 29 (23.8) | 9 (16.4) | 38 (21.5) |
| Cardiac abnormalities/cardiovascular disease | 17 (13.9) | 11 (20.0) | 28 (15.8) |
| Sleep disorders | 17 (13.9) | 10 (18.2) | 27 (15.3) |
| Diabetes mellitus | 17 (13.9) | 8 (14.5) | 25 (14.1) |
| Polyneuropathy/neuropathy | 15 (12.3) | 7 (12.7) | 22 (12.4) |
| Fatigue | 14 (11.5) | 5 (9.1) | 19 (10.7) |
| Orthostatic dysregulation | 13 (10.7) | 5 (9.1) | 18 (10.2) |
| Chronic gastrointestinal disease | 12 (9.8) | 2 (3.6) | 14 (7.9) |
| Any malignancy | 5 (4.1) | 6 (10.9) | 11 (6.2) |
| Chronic pulmonary disease | 5 (4.1) | 3 (5.5) | 8 (4.5) |
| Skin disease | 1 (0.8) | 3 (5.5) | 4 (2.3) |
| Chronic renal disease or insufficiency | 1 (0.8) | 3 (5.5) | 4 (2.3) |
| Chronic liver disease or insufficiency | 1 (0.8) | 0 (0) | 1 (0.6) |
| Other | 45 (36.9) | 20 (36.4) | 65 (36.7) |
| Multiple entries possible |  |  |  |

**Supplemental Table 2.** APD classification by Delphi method vs. APD classification by physician

|  | | **APD classification by Delphi method** | | | |
| --- | --- | --- | --- | --- | --- |
| **Delphi criteria for APD classification** | **Physician's judgement** | **Non-APD n (%)** | **APD n (%)** | **Cohen’s Kappa**  **[95%-CI]** | |
| 1. Moderate/severe troublesome motor fluctuations | APD | 62 (50.8) | 60 (49.2) | 0.315 | [0.213; 0.417] |
|  | Non-APD | 43 (95.6) | 2 (4.4) |  |  |
| 2. At least 2 hours of the waking day with “off” symptoms | APD | 74 (60.7) | 48 (39.3) | 0.166 | [0.066; 0.266] |
|  | Non-APD | 39 (84.8) | 7 (15.2) |  |  |
| 3. Moderate/severe night time sleep disturbances | APD | 78 (63.9) | 44 (36.1) | 0.064 | [-0.045; 0.172] |
|  | Non-APD | 35 (72.9) | 13 (27.1) |  |  |
| 4. At least 2 hours of the day with troublesome dyskinesia | APD | 97 (79.5) | 25 (20.5) | 0.113 | [0.054; 0.172] |
|  | Non-APD | 46 (97.9) | 1 (2.1) |  |  |
| 5. NMS fluctuations | APD | 55 (45.1) | 67 (54.9) | 0.259 | [0.134; 0.383] |
|  | Non-APD | 42 (76.4) | 13 (23.6) |  |  |
| 6. “Off” time at least every 3 hours | APD | 86 (70.5) | 36 (29.5) | 0.158 | [0.075; 0.241] |
|  | Non-APD | 51 (92.7) | 4 (7.3) |  |  |
| 7. At least 5 times daily oral levodopa dosing | APD | 54 (44.3) | 68 (55.7) | 0.344 | [0.228; 0.460] |
|  | Non-APD | 47 (87.0) | 7 (13.0) |  |  |
| 8. Moderate/severe of limitation of ADL capacity | APD | 62 (50.8) | 60 (49.2) | 0.242 | [0.126; 0.357] |
|  | Non-APD | 44 (81.5) | 10 (18.5) |  |  |
| 9. Falls most of the time/all the time | APD | 113 (92.6) | 9 (7.4) | 0.039 | [0.012; 0.067] |
|  | Non-APD | 42 (100.0) | 0 (0.0) |  |  |
| 10. Moderate/severe dementia | APD | 110 (90.2) | 12 (9.8) | -0.001 | [-0.057; 0.056] |
|  | Non-APD | 36 (90.0) | 4 (10.0) |  |  |
| 11. Moderate/severe psychosis | APD | 115 (94.3) | 7 (5.7) | 0.029 | [0.006; 0.051] |
|  | Non-APD | 39 (100.0) | 0 (0.0) |  |  |
| Overall APD classification | APD | 12 (9.8) | 110 (90.2) | 0.243 | [0.077; 0.410] |
|  | Non-APD | 13 (31.0) | 29 (69.0) |  |  |
